# Improving Protein Quantification with SERS Superspectra and Machine Learning

**DOI:** 10.1021/acsomega.6c00157

**Published:** 2026-02-04

**Authors:** Jiaheng Cui, Chenyao Feng, Xulan Chen, Yanjun Yang, Pengju Yin, Yiping Zhao

**Affiliations:** † School of Electrical and Computer Engineering, College of Engineering, 1355 The University of Georgia, Athens, Georgia 30602, United States; ‡ School of Mathematics and Physics, 117798 Hebei University of Engineering, Handan, Hebei 056038, China; § Department of Biochemistry and Molecular Biology, Franklin College of Arts and Sciences, The University of Georgia, Athens, Georgia 30602, United States; ∥ Department of Physics and Astronomy, Franklin College of Arts and Sciences, The University of Georgia, Athens, Georgia 30602, United States

## Abstract

Quantitative protein analysis by surface-enhanced Raman spectroscopy (SERS) remains challenging due to weak and heterogeneous protein adsorption on plasmonic surfaces. Here, we introduce a superspectra-guided SERS framework that leverages chemically distinct interaction environments to enhance quantitative performance. Silver nanorod (AgNR) substrates were functionalized with cysteamine (CM), cysteine (CN), and 6-mercapto-1-hexanol (MCH), together with unmodified (B) AgNRs, to create surfaces that probe complementary aspects of protein–surface interactions through charge- and chemistry-dependent binding. Using bovine serum albumin (BSA) as a model protein, we systematically constructed superspectra by concatenating SERS signals from all single-, pairwise-, triple-, and four-surface combinations and evaluated their performance using support vector regression (SVR) and random forest regression (RFR). Our results reveal that superspectra must be constructed selectively: single-substrate spectra lack sufficient chemical diversity, and superspectra incorporating all four surfaces often degrade accuracy due to noninformative or conflicting features, particularly those introduced by MCH. In contrast, superspectra derived from complementary surface chemistries, especially the CM&CN pair or the B&CM&CN triplet, yield markedly improved quantitative predictions. RFR consistently outperformed SVR, demonstrating superior robustness for integrating chemically heterogeneous spectral inputs. This work establishes, for the first time, design principles for constructing effective superspectra for protein SERS and highlights the importance of analyte–surface interaction complementarity in enabling accurate, scalable protein quantification.

## Graphical abstract

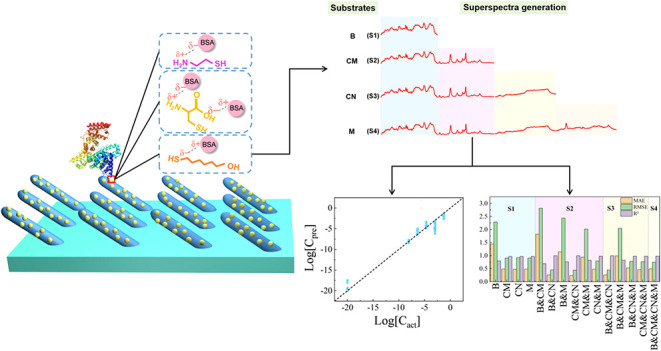

## I Introduction

Accurate protein quantification is fundamental to biomedical research, providing critical insights into physiological and pathological processes, enzyme kinetics, signaling networks, and drug development pathways.^1–3^ With the rise of personalized medicine, reliable protein measurement has become even more essential for validating biomarkers and tailoring targeted therapies.^4,5^ Traditional techniques for protein detection, such as enzyme-linked immunosorbent assay (ELISA), mass spectrometry (MS), and Western blotting, have made substantial contributions but still face challenges, including low sensitivity, limited specificity, and susceptibility to matrix effects in complex biological samples.^6–8^ These limitations have prompted the search for alternative analytical tools with higher sensitivity, specificity, and multiplexing capabilities.

Surface-enhanced Raman spectroscopy (SERS) has emerged as an attractive platform for protein detection due to its label-free molecular specificity and ability to amplify Raman scattering signals by several orders of magnitude via localized surface plasmon resonances.^9^ These properties have enabled numerous protein-related SERS applications, from structural characterization to disease biomarker detection.^10^ Nevertheless, quantitative protein SERS remains challenging. Large, globular proteins often exhibit weak, orientation-dependent adsorption on bare metal surfaces, producing variable enhancement and poor reproducibility.^11^ Furthermore, their dense and overlapping vibrational signatures complicate peak-based interpretation.^12^ To improve adsorption and stabilize protein–substrate interactions, researchers have developed functional surface coatings, including various thiolated self-assembled monolayers (SAMs) such as cysteamine (CM),^13,14^ cysteine (CN),^15^ 6-mercapto-1-hexanol (MCH),^16^ etc., that modulate charge, hydrophobicity, and chemical affinity.^17–21^ While these strategies can improve spectral quality, excessive or inappropriate surface modifiers may introduce background Raman features, sterically hinder adsorption, or perturb protein orientation in ways that suppress key vibrational modes.^22,23^

Machine learning (ML) techniques have, therefore, become increasingly valuable for interpreting high-dimensional protein SERS spectra. ML has enabled discrimination of structurally similar proteins, identification of biochemical signatures, and quantification of disease biomarkers.^24–27^ More recently, several studies have attempted to improve analytical performance by combining spectra acquired under different sensing conditions, producing what is often termed a superspectrum. Such approaches concatenate spectra from multiple receptors, substrates, or measurement modalities to expand chemical diversity and enrich the information content. Superspectra have been successfully applied to gas sensing, volatile-organic-compound profiling, flavor analysis, and environmental contaminants.^28–34^ These works consistently report that combining complementary interaction environments, e.g., receptors targeting hydrogen bonding, hydrophobicity, or electrostatic interactions, improves classification accuracy and sometimes regression performance. However, in nearly all cases, superspectra are constructed using a fixed set of receptors, and performance improvements are reported without a detailed analysis of why certain combinations work, whether some combinations degrade performance, or how surface chemistry influences the underlying information content.

Notably, none of the existing superspectra literature examines proteins, which pose fundamentally different challenges from small molecules due to their size, charge heterogeneity, conformational flexibility, and complex adsorption behavior. Moreover, prior studies do not address whether superspectra can fail, whether certain surface chemistries contribute noninformative or conflicting features that degrade quantitative prediction. Thus, despite significant promise, the principles governing effective superspectra construction remain unclear, particularly for protein sensing. To address this gap, we selected bovine serum albumin (BSA) as a model system. BSA is a structurally stable, highly soluble, and extensively characterized protein widely used in biochemical research.^35,36^ Its heterogeneous distribution of charged, hydrophobic, and aromatic residues makes its adsorption highly sensitive to surface chemistry, providing an ideal benchmark for evaluating how functionalized SERS substrates encode complementary interaction modes into superspectra.

Here, we introduce a superspectra-guided SERS framework for quantitative protein analysis using silver nanorod (AgNR) substrates functionalized with CM, CN, and MCH, as well as bare AgNRs (B). By systematically generating superspectra from all pairwise, triple, and four-way combinations of these substrates, and analyzing their performance using support vector regression (SVR) and random forest regression (RFR), we address a critical gap left by prior superspectra research: Superspectra cannot be arbitrarily constructed; only specific, analyte-relevant combinations yield improved quantitative performance. Our results demonstrate that (1) single-substrate spectra lack sufficient chemical diversity for accurate BSA quantification. (2) Not all superspectra improve performance; inclusion of MCH often reduces predictive accuracy due to noninformative or interfering spectral contributions. (3) Superspectra derived from complementary surface charge environments (e.g., CM&CN or B&CM&CN) significantly enhance ML regression accuracy by capturing diverse protein–surface interaction modes. (4) RFR consistently outperforms SVR, indicating that nonlinear ensemble models are better suited for integrating chemically heterogeneous spectral inputs. Together, these findings establish that superspectra design must be guided by analyte–surface interaction chemistry, not simply by data concatenation.

## II Experiment

### II.I Materials and Methods

Silver and titanium for deposition were acquired from Kurt J. Lesker, while glass microscope slides for AgNR substrate preparation were purchased from Thermo Fisher Scientific. The chemicals MCH, CM, and CN, as well as protein BSA, were all sourced from Sigma-Aldrich. For the creation of PDMS gel wells, the Sylgard 184 Silicone Elastomer Base and Curing Agent were obtained from Dow.

### II.II Silver Nanorod (AgNR) Arrays Fabrication

AgNR arrays were fabricated on a clean 0.5-in. × 0.5-in. glass slides using oblique angle deposition, as described in detail in References.^37,38^ Figure 1A shows the geometry of the AgNR array SERS substrate, and Figure S1 of the Supporting Information (SI) presents a representative electron micrograph. This fabrication method consistently produces tilted Ag nanorods with nanometer-scale interrod gaps (typically 100–200 nm) and sharp tips that act as dense SERS hot spots. The structural parameters, such as rod length, tilt angle, and gap spacing, are highly uniform across each substrate and between fabrication batches, resulting in reproducible SERS performance. Prior studies have reported substrate-to-substrate signal variation below ∼15% and enhancement factors in the 10^6^–10^9^ range. Additional details on substrate characteristics and long-term reproducibility can be found in our recent review summarizing two decades of AgNR substrate development.^39^ Before surface modification, all substrates were plasma cleaned, and a poly­(dimethylsiloxane) (PDMS) layer containing four wells (2 × 2 arrangement, each 2 mm radius and 1 mm depth) was molded onto the surface to isolate functionalization and measurement regions.^40^

**Figure 1:**
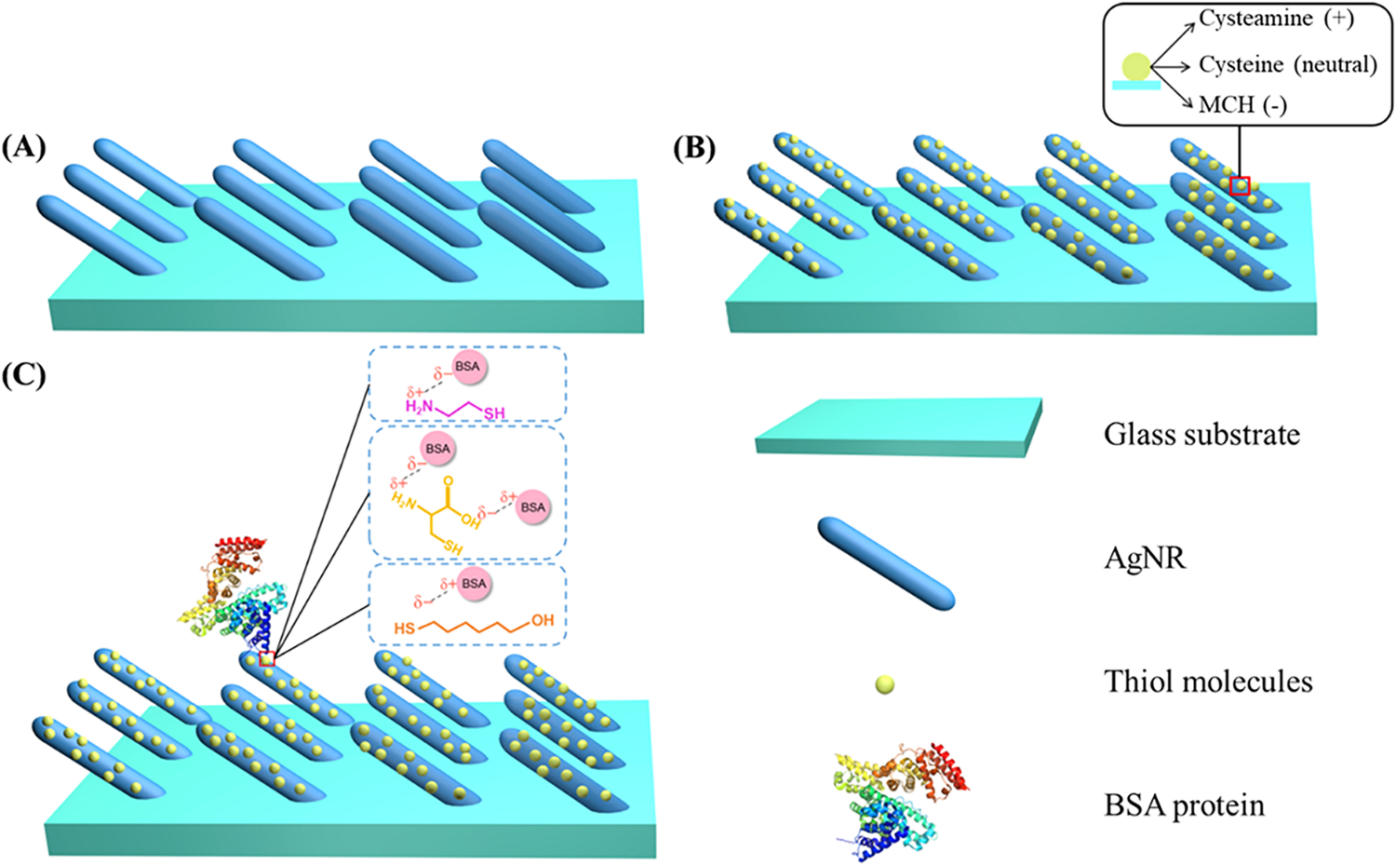
Schematic illustration of protein adsorption on functionalized AgNR substrates. (A) Silver nanorods (AgNRs) deposited on a glass substrate. (B) Formation of self-assembled monolayers of thiol molecules on the AgNR surface. (C) Electrostatic interaction between charged sites on BSA molecules and the surface-modifying thiol monolayers, facilitating selective protein adsorption based on surface charge.

### II.III Functionalization of AgNR Substrates

Three thiol molecules, CM, CN, and MCH, were used to functionalize the AgNR substrates, as shown in Figure 1B, to impart different surface charges and modulate the BSA protein adsorption. CM creates a positively charged surface via its terminal amine group (−NH_3_^+^), CN is zwitterionic, containing both –NH_3_^+^ and −COO^–^ groups, and MCH forms a negatively charged surface due to its hydroxyl group (−OH). As shown in Figure 1C, these surface charges can influence how BSA proteins interact with the substrate through electrostatic interactions:^41,42^ On CM-modified surfaces, negatively charged residues in BSA (e.g., aspartic acid and glutamic acid) are preferentially attracted; on CN-modified surfaces, both positive and negative residues can interact due to the balanced charge of the surface; and on MCH-modified surfaces, positively charged residues (e.g., arginine and lysine) tend to bind.

For CM functionalization, substrates were immersed in 2 mL of a 300 μM CM solution for 1 h, rinsed with deionized (DI) water for 60 s, and dried under nitrogen. CN and MCH functionalizations were performed using 1 mM solutions prepared in 10 mL volumes; substrates were submerged in 2 mL of each solution for 1 h, thoroughly rinsed with deionized water, and dried under nitrogen flow.

### II.IV BSA Solution Preparation

SA stock solutions were prepared in DI water and diluted to five concentrations: 0.05, 1 × 10^–3^, 2 × 10^–5^, 4 × 10^–7^, 8 × 10^–9^ mg/mL. For each functionalized substrate, 20 μL of a BSA solution was dispensed into each PDMS well and allowed to dry overnight to ensure complete evaporation prior to SERS measurements.

### II.V SERS Measurements

SERS measurements were performed on a Renishaw Raman microscope equipped with a 785 nm laser, using 10% laser power, a 5× objective, and a 10 s acquisition time per spectrum. To ensure adequate statistical sampling, 2–4 AgNR substrates (5–15 PDMS wells in total) were used for each type of surface functionalization, resulting in 50–60 spectra per concentration per surface modification. Mapping points were distributed across the PDMS wells on both functionalized and bare AgNR substrates. Although minor variations in well usage occurred due to experimental scheduling and substrate availability, the total number of spectra acquired per condition was kept consistent to ensure comparability. Additionally, 30–35 reference spectra (modifier-only) were collected for each substrate type. Reference spectra were obtained prior to BSA exposure.

### II.VI Spectrum Preprocessing and Analysis

All SERS spectra were preprocessed using a standardized three-step workflow applied uniformly across all substrate types and BSA concentrations to ensure consistency and reproducibility: (1) To standardize wavenumber sampling, all spectra were interpolated to a uniform 1 cm^–1^ grid from 401–1799 cm^–1^, yielding 1,399 data points per spectrum. (2) A Gaussian–Lorentzian fitting (GLF) method^43^ was first used to model and subtract the smooth baseline envelope, followed by adaptive iteratively reweighted penalized least-squares (airPLS)^44^ to refine residual baseline variations. (3) Each spectrum was mean-normalized by dividing all intensities by the spectral mean. All preprocessing steps were performed using SpectraGuru (https://spectraguru.org/),^45^ our automated online spectral processing platform, ensuring consistent parameter application and full reproducibility of the analysis pipeline.

### II.VII Superspectra Generation

Superspectra were generated by concatenating the above preprocessed SERS spectra collected from different surface-modified AgNR substrates to create feature vectors that integrate complementary chemical information for ML analysis. As shown in Figure 2, spectra from the four substrate types (B, CM, CN, MCH) were first grouped by identical BSA concentration, ensuring that all spectra combined into a superspectrum represent the same adsorption condition. The four data sets were arranged alphabetically (B → CM → CN → MCH) to maintain a consistent and reproducible ordering; this ordering has no impact on model performance because ML algorithms operate on fixed-length feature vectors rather than ordered sequences. Superspectra of different sizes were then constructed by concatenating spectra from one substrate (S1), pairs (S2), triplets (S3), or all four substrates (S4). For each combination, one spectrum was randomly selected from each substrate’s data set at the same BSA concentration, and their intensity arrays were appended to form a single extended vector. For example, concatenating B and CM spectra (each containing 1,399 data points after preprocessing) produces a 2,798-point superspectrum. This procedure was repeated to generate all of the possible combinations. The number of superspectra generated for each configuration (S1 to S4) depends on the number of spectra available for each substrate at a given concentration; details can be found in Section S1 of SI.

**Figure 2:**
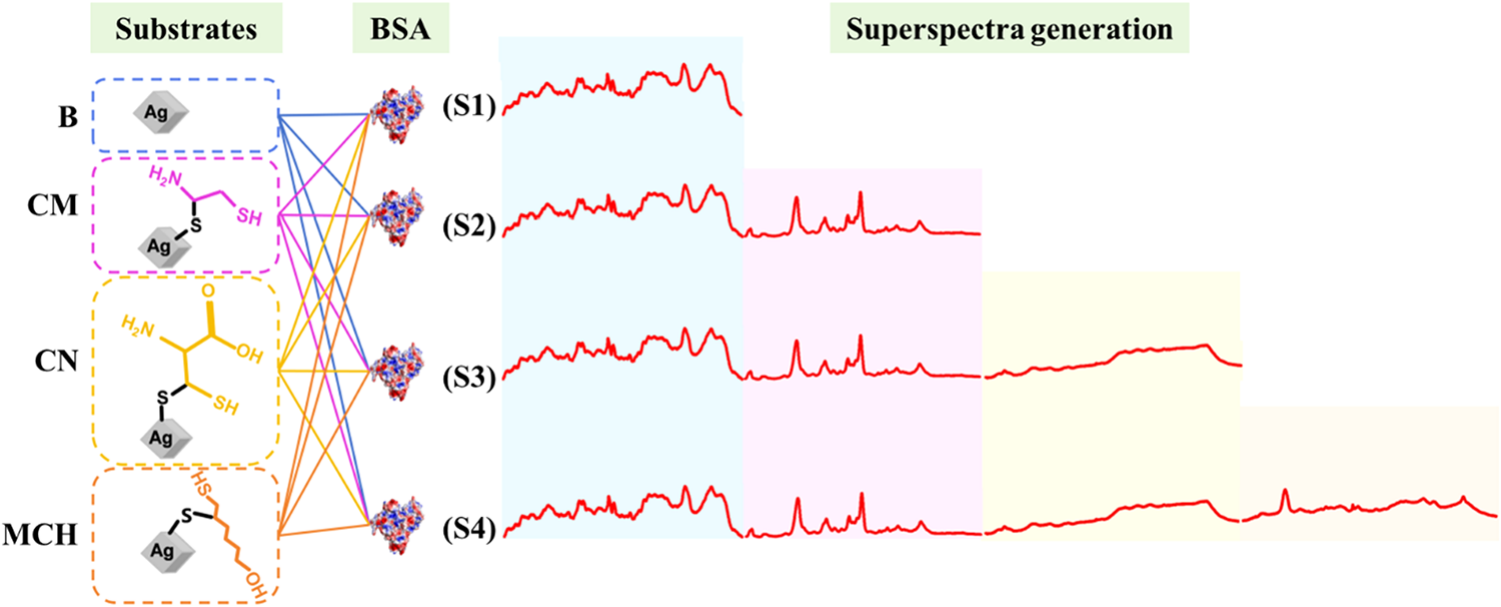
Schematic illustration of superspectra generation. SERS spectra from individual surface-modified substrates—bare (B), cysteamine (CM), cysteine (CN), and MCH, are arranged in alphabetical order and concatenated to form superspectra.

Although concatenation creates junctions between spectral segments, these do not affect model performance because (1) each spectrum is normalized before concatenation, (2) the junctions occur at low-signal boundary regions of the spectra, and (3) RFR and SVR treat spectra as high-dimensional feature vectors and are insensitive to local discontinuities. Empirically, superspectra consistently outperform single-substrate spectra, confirming that added chemical information outweighs any computational artifacts.

### II.VIII Classification Models

To assess whether SERS spectra from different surface modifications are distinguishable (a necessary condition for constructing meaningful superspectra), we performed classification analysis prior to regression modeling. The full data set was randomly divided into training and test sets using stratified 2:1 sampling, ensuring that each substrate–concentration group was proportionally represented in both subsets and preventing data leakage.

Two standard machine learning classifiers were used: Support Vector Machine (SVM)^46,47^ and Random Forest (RF).^48–50^ Both were implemented with scikit-learn’s default settings, as classification in this context is a relatively simple task compared to concentration regression. For SVM, a linear-kernel (kernel = “linear”, *C* = 1.0) was selected due to the high dimensionality of the spectral feature space (1399–5596 features), where linear separation is often effective. For the RF classifier, we used default parameters (n_estimators = 100, max_depth = None, min_samples_split = 2, min_samples_leaf = 1).

Default parameters were intentionally used because classification merely verifies that each surface modification generates distinct spectral patterns—an expectation grounded in their differing surface chemistries. This task is substantially less complex than quantitative regression across 5 orders of magnitude. In contrast, the regression models used later in this study underwent full hyperparameter tuning (grid search with cross-validation), as accurate concentration prediction is far more sensitive to model parametrization. This tiered approach allows computational resources to be focused on the central analytical goal: quantitative prediction of protein concentrations using superspectra.

### II.IX Regression Models for Concentration Prediction

Quantitative prediction of the BSA concentration from SERS spectra and superspectra was performed using Support Vector Regression (SVR) and Random Forest Regression (RFR). Unlike the classification models used earlier, the regression variants optimize continuous outputs and therefore require systematic hyperparameter tuning. Regression analysis was conducted for all substrate combinations (S1–S4) using the data sets summarized in Table S1 and the train/test splitting strategy described in Section 2.7.

Because the regression task spans 5 orders of magnitude in concentration (8 × 10^–9^ to 0.05 mg/mL), both models underwent 5-fold cross-validated grid search on the training set to identify optimal hyperparameters. The parameter ranges tested (Table S2) included linear, polynomial, and RBF kernels with multiple values of C and ϵ for SVR, and variations in the number of trees, maximum depth, and node-splitting criteria in RFR. For each substrate combination, the final hyperparameters were selected based on the lowest mean absolute error (MAE) averaged across validation folds, ensuring generalization rather than overfitting. Grid search consistently identified SVR with an RBF kernel (*C* = 100, ϵ = 0.1) and RFR with n_estimators = 100 and max_depth = None as the best-performing configurations. These optimized models were retrained on the full training set and evaluated on the held-out test set, which was not used during hyperparameter tuning.

Model performance was quantified using three standard regression metrics: MAE (average magnitude of prediction errors), Root Mean Square Error (RMSE, an error measure that penalizes larger deviations), and the coefficient of determination (*R* ^2^, proportion of variance in concentration explained by the model). Lower MAE and RMSE and higher *R* ^2^ (closer to 1) indicate a stronger predictive performance.

All analyses were performed using Python 3.11.5 and scikit-learn 1.4.1.^51^

## III Results and Discussion

### III.I Spectra Features and Discussions

Figure 3 presents the average normalized SERS spectra of BSA adsorbed on bare (B), CM-, CN-, and MCH-modified substrates. Each curve represents the mean of multiple spectra, and averaging was used to highlight robust spectral features while reducing noise (see variability analysis in Figure S2). Peak positions and vibrational assignments are summarized in Table 1 and compared with the literature values.

**Figure 3:**
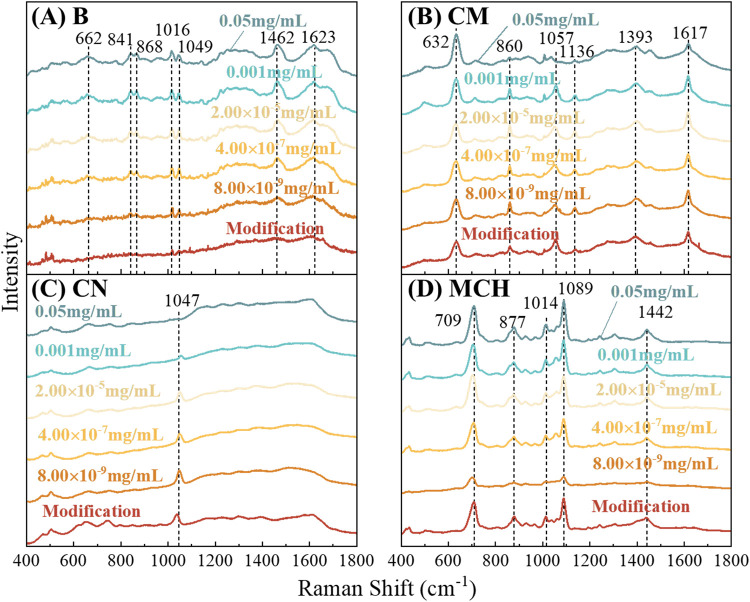
Average normalized SERS spectra of BSA adsorbed on AgNR substrates with four surface chemistries: (A) bare (B), (B) cysteamine (CM), (C) cysteine (CN), and (D) MCH. In each panel, the red curve labeled “Modification” represents the substrate without BSA, serving as a reference to identify modifier-specific vibrational features. The remaining spectra show responses at different BSA concentrations (8 × 10^–9^, 4 × 10^–7^, 2 × 10^–5^, 0.001, and 0.05 mg/mL, from bottom to top).

**Table 1:** Summary of SERS Peaks from Figure 3 and Literature Comparison for BSA and Three Thiol Molecules (Cysteamine, Cysteine, and MCH)

peak (cm^–1^)	lit.	bare	cysteamine	cysteine	MCH
632	628–653 C–S stretch (gauche)^52^		√		
662	ν(C–S), Tyr^53^	√			
709	712–743 C–S stretch (trans)^54^				√
841	Tyr^53^	√			
860	865.5 ^55^		√		
868	Tyr^53^	√			
877	870 C–C–C stretching^54^				√
1014	1011 C–C–C stretching^54^				√
1016	1004 R breathing^53^	√			
1047	1058 C α–N stretching^56^			√	
1049	ν(−C–N), Phe^53^	√			
1057	1045, 1047, 1064 ^55^		√		
1089	1083 C–O stretching^54^				√
1136	1126.5 ^55^		√		
1393	1436 ^57^		√		
1442	1436 C–H bending^54^				√
1462	δ(C–H), δ(CH2/CH3)^53^	√			
1617	1631 ^57^		√		
1623	amino acids^53^	√			

Bare substrate (Figure 3A) produces a minimal background signal, allowing BSA peaks to be clearly identified. Upon BSA adsorption, characteristic BSA peaks are observed at Δν = 662, 841, 868, 1016, 1049, 1462, and 1623 cm^–1^, consistent with literature assignments:^53^ C–S stretching vibrations or tyrosine (Tyr) side-chain modes at Δν = 662 cm^–1^, Tyr aromatic ring stretching at Δν = 841 and 868 cm^–1^, phenylalanine (Phe) ring breathing at Δν = 1016 cm^–1^, C–N stretching in Phe at Δν = 1049 cm^–1^, aliphatic CH_2_/CH_3_ C–H bending vibrations at Δν = 1462 cm^–1^, and amide-related modes at Δν = 1623 cm^–1^. These peaks serve as the reference spectral fingerprints for protein adsorption.

CM-modified substrates (Figure 3B) introduces a strong spectral contribution from the modifier itself, with identifiable CM peaks at Δν = 632, 860, 1057, 1136, 1393, and 1617 cm^–1^.^52,55,57^ Compared with the bare substrate, several BSA bands shift or change in intensity due to electrostatic attraction between BSA’s negatively charged residues and CM’s terminal – NH_3_^+^ group:^53^ Tyr mode at Δν = 860 cm^–1^ appears shifted and weakened relative to the 868 cm^–1^ band on bare AgNRs; Phe C–N stretching at Δν = 1057 cm^–1^ shows altered intensity, reflecting residue reorientation toward the positively charged surface; and amide-related vibrations at Δν = 1617 cm^–1^ are enhanced, suggesting increased BSA adsorption density. However, some BSA peaks visible on bare AgNRs (such as Δν = 662, 841, 1016, and 1462 cm^–1^) are diminished or absent. This is attributed not only to protein reorientation driven by strong electrostatic interactions but also to the masking effect of the intense CM Raman bands. Together, these features confirm both effective CM functionalization and the charge-mediated modulation of BSA adsorption.

The CN-modified substrate (Figure 3C) exhibits a distinct marker peak at Δν = 1047 cm^–1^, corresponding to the C-α-N stretching vibration in CN.^56^ This confirms the successful SAM formation. Importantly, the intensity of this peak decreases with increasing BSA concentration, reflecting competitive adsorption: BSA progressively displaces CN molecules from the surface. Compared with the bare substrate, BSA signals on CN-modified surfaces are overall weaker. CN’s zwitterionic character provides balanced −NH_3_^+^/–COO^–^ interactions, resulting in more uniform but less tightly bound protein adsorption. Thus, although BSA vibrational modes appear, they are reduced in intensity, and the net spectral profile differs substantially from those of both bare and CM-modified substrates.

The MCH-modified substrate (Figure 3D) is dominated by modifier-specific peaks at Δν = 709, 877, 1014, 1089, and 1442 cm^–1^, corresponding to C–S stretching (trans), C–C–C stretching, C–O stretching, and alkyl-chain bending modes.^54^ These features overshadow the BSA signals. Most BSA characteristic peaks seen on bare AgNRs (Δν = 662, 841, 1049, 1462, and 1623 cm^–1^) are absent or severely suppressed. Although peaks at 877 and 1014 cm^–1^ appear near BSA-relevant frequencies, they are clearly dominated by MCH and cannot be interpreted as protein signatures. This strong suppression arises from unfavorable adsorption: MCH’s negatively charged −OH-terminated surface repels negatively charged BSA residues (e.g., Asp and Glu), leading to weak or distant adsorption orientations that place most amino acids outside the SERS enhancement zone.

Taken together, the results in Figure 3 show that although the core vibrational features of BSA can be detected on all substrates, the local interfacial environment created by each surface modifier selectively enhances, suppresses, or shifts specific Raman modes. These substrate-dependent variations arise from differences in electrostatic interactions, chemical resonance effects, and adsorption-induced changes in the protein conformation or orientation. Recognizing and understanding these effects are essential both for interpreting SERS spectra accurately and for designing functionalized substrates that enable sensitive, reliable protein detection.

Building on this spectral analysis, we next evaluated whether SERS peak intensities could be used for the traditional calibration-curve–based quantification of BSA. A common approach is to track the intensity of a characteristic vibrational mode *I*_Δν_ against BSA concentration *C*_BSA_, as shown in Figure 4. In principle, BSA-related peaks *I*_Δν_ should increase monotonically with higher *C*_BSA_ due to greater adsorption, whereas peaks associated with surface modifiers should remain constant or decrease because of competitive displacement. However, the experimental data reveal that monotonic behavior is rarely observed.

**Figure 4:**
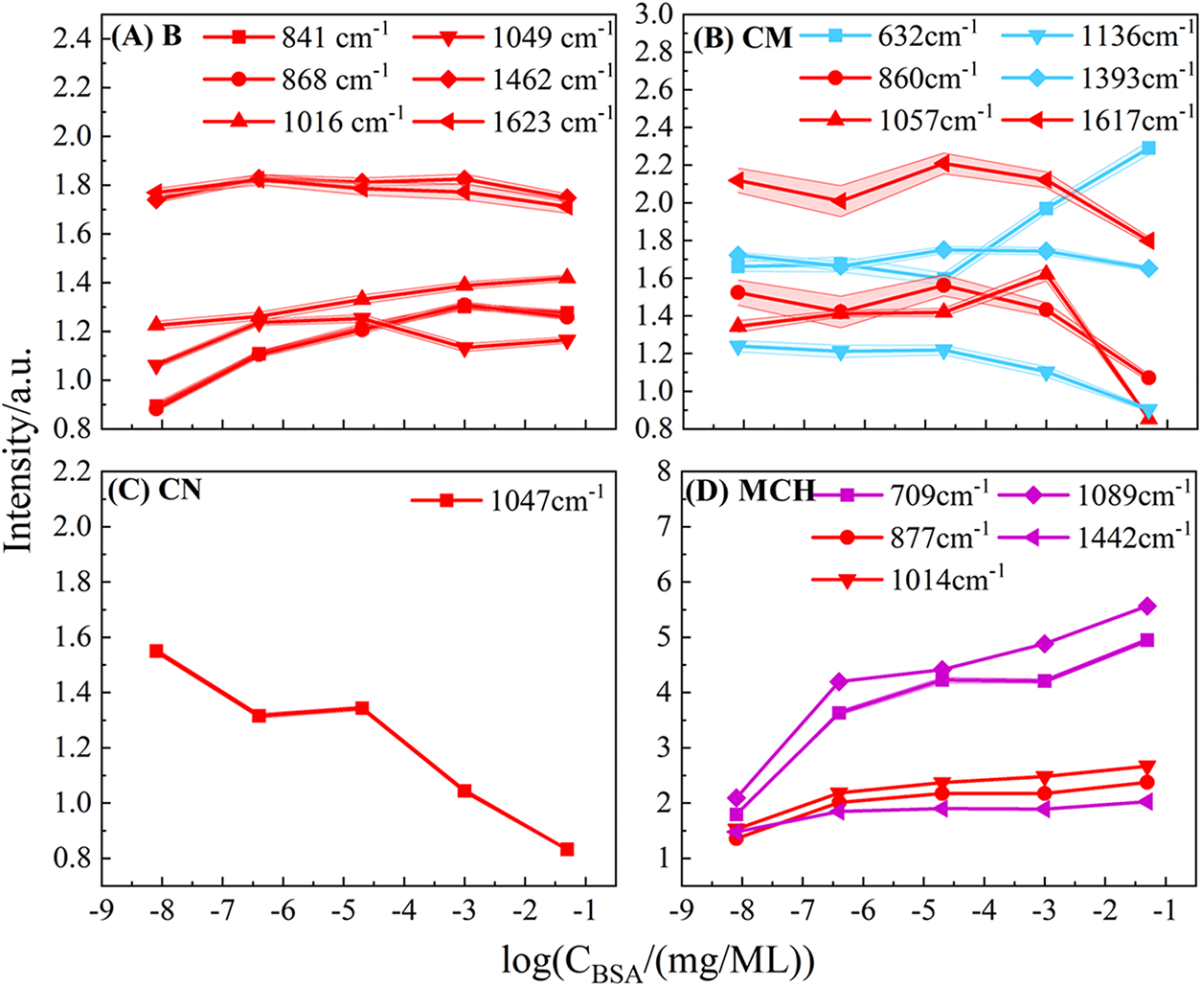
Intensity–concentration trends for major Raman peaks extracted from BSA SERS spectra on AgNR substrates with four surface chemistries: (A) bare (B), (B) cysteamine (CM), (C) cysteine (CN), and (D) MCH. Each colored line represents the intensity of a specific vibrational peak across the five BSA concentrations. Red-labeled peaks denote modes primarily associated with the BSA.

For the bare substrate as shown in Figure 4A, characteristic BSA peaks (e.g., Δν = 662, 841, 868, 1016, 1049, 1462, and 1623 cm^–1^) do not vary systematically with concentration; intensities fluctuate rather than increase smoothly. For the CM-modified substrate (Figure 4B), BSA peaks at Δν = 860, 1057, and 1617 cm^–1^ again show no clear monotonic trend. The CM-specific 1136 cm^–1^ peak decreases with increasing BSA concentration, consistent with competitive adsorption, but this behavior adds complexity rather than improving quantification. For the CN-modified substrate (Figure 4C), the CN characteristics peak at Δv = 1047 cm^–1^ decreases with increasing BSA concentration, yet exhibits plateaus at intermediate concentrations, reflecting irregular displacement dynamics and weakening its usefulness for calibration. For the MCH-modified substrate (Figure 4D), several MCH-derived peaks at Δν = 709, 877, 1014, and 1089 cm^–1^ increase with concentration, but BSA-specific modes (red-labeled) remain weak and show no consistent trend because BSA adsorption is strongly suppressed.

### III.II Machine Learning Analysis

As shown in Figure 4, BSA peak intensities on all four substrates exhibit nonmonotonic and inconsistent trends with concentration, making traditional peak-based calibration curves unreliable. These irregular patterns arise from competitive adsorption, surface-site saturation, and substrate-specific protein interactions, all of which alter the spectral features in nonlinear ways. ML is therefore required to extract quantitative information from the full spectral profile rather than relying on individual peaks. Our ML workflow consists of two complementary components: (1) Classification, to verify whether the four substrate modifications (B, CM, CN, MCH) produce distinct and complementary spectral signatures. Without this distinction, concatenating spectra into superspectra would not increase chemical information content. (2) Regression, the central analytical task, is used to quantitatively predict BSA concentration from single-substrate spectra and from all superspectra combinations. The complete methodologies are detailed in Sections 2.8 (Classification) and II.IX (Regression).

Both SVM and RF classifiers were trained to distinguish the spectra (S1) from the four modified substrates. Both models achieved 100% accuracy on the held-out test set (see Figure S3), confirming that each surface chemistry yields spectrally unique signatures. These differences arise from how each modifier alters the local chemical environment and influences BSA adsorption and orientation. This perfect separation validates the theoretical basis for superspectra construction: combining spectra from multiple modifications introduces genuinely complementary chemical information rather than redundant features.

Having confirmed that each surface modification produces spectrally distinct features, we next evaluated the ability of ML models to quantitatively predict the BSA concentration. We performed regression using both single-substrate spectra (S1) and different combinations of superspectra constructed from two, three, or four substrates (S2–S4). For each case, we trained SVR and RFR models and optimized all hyperparameters using grid search. Generally, the RFR models gave better performance than the SVR models.

Figure 5A shows the best-performing single-substrate (S1) model, obtained using CN spectra alone (MAE = 0.477, RMSE = 0.916, and *R* ^2^ = 0.966). Although many predictions fall near the diagonal, noticeable deviations highlight the limited chemical information available from any single surface, establishing the need for superspectra constructed from multiple, complementary substrates. We therefore evaluated regression performance using S2–S4 superspectra. Table S3 summarizes the optimal performance for all S1–S4 cases by RFR. Importantly, improvements do not result from simply concatenating more spectra; rather, the chemical complementarity of the substrates determines prediction quality.

**Figure 5:**
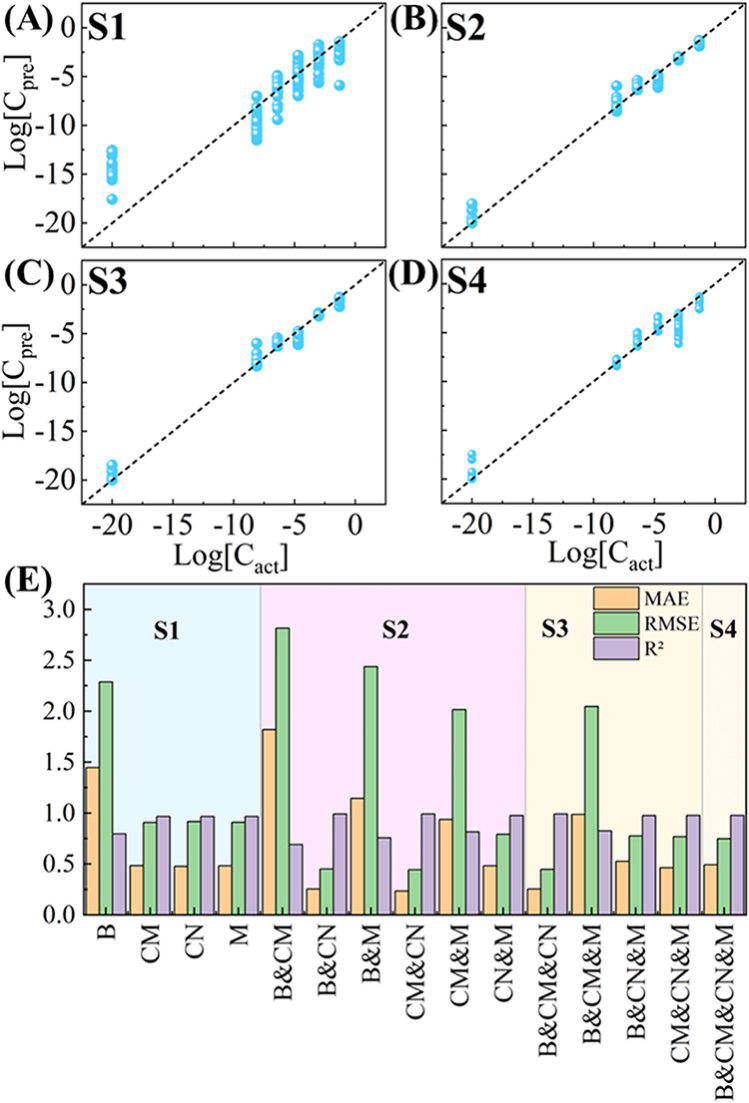
Predicted vs actual BSA concentrations for the best-performing RFR regression models in each superspectra category: (A) CN for S1; (B) CM&CN for S2; (C) B&CM&CN for S3; and (D) B&CM&CN&MCH (the only S4 model). (E) Bar plots summarizing MAE, RMSE, and R^2^ for all superspectra configurations obtained from optimal RFR models.

The CM&CN superspectrum (Figure 5B) yields the best S2 performance (MAE = 0.235, RMSE = 0.444, R^2^ = 0.993). CM’s positive charge enhances interactions with acidic BSA residues, while CN’s zwitterionic character interacts with both positive and negative residues. This complementary interaction profile produces a richer, nonredundant feature set that strongly boosts regression accuracy. Other S2 combinations highlight the importance of substrate pairing. For example, B&CM performs poorly (MAE = 1.820, RMSE = 2.816, *R* ^2^ = 0.690) because both surfaces preferentially bind negatively charged residues, leading to redundant spectral information and reduced generalizability.

Adding the bare substrate to the complementary CM&CN pair (Figure 5C) produces B&CM&CN, one of the strongest performers overall (MAE = 0.254, RMSE = 0.446, *R* ^2^ = 0.993). The bare substrate provides a neutral adsorption baseline that mitigates variabilities introduced by charged surfaces, improving robustness without introducing redundancy. In contrast, S3 combinations including MCH, such as B&CM&MCH, perform substantially worse (MAE = 0.986, RMSE = 2.047, *R* ^2^ = 0.825). MCH’s negative surface repels acidic BSA residues, contributing little analyte-specific information and diluting the benefits of the more informative substrates.

The full S4 combination B&CM&CN&MCH (Figure 5D) exhibits intermediate performance (MAE = 0.494, RMSE = 0.748, *R* ^2^ = 0.978). Although it incorporates the largest amount of spectral information, its accuracy is slightly lower than that of the best S2 and S3 combinations. Notably, its performance is worse than most S2 and S3 combinations that exclude MCH, yet better than all combinations that include MCH, reflecting the detrimental impact of the MCH substrate. Because MCH contributes weak or conflicting spectral features due to poor BSA adsorption, its inclusion offsets the advantages of otherwise complementary substrates. These results reinforce a central conclusion of this study: superspectra quality depends on substrate compatibility rather than on maximizing the number of concatenated spectra.

Table S3 provides the detailed MAE, RMSE, and *R* ^2^ values for all S1–S4 combinations, and Figure 5E visualizes these results using a bar plot. While these numerical values allow detailed assessment, the multiparameter nature of the comparison makes it difficult to visually identify overall performance trends. For instance, the B substrate alone performs poorly (MAE = 1.443, RMSE = 2.298, *R* ^2^ = 0.795), whereas the S3 B&CM&CN superspectrum shows dramatically improved accuracy (MAE = 0.254, RMSE = 0.446, *R* ^2^ = 0.993). Similarly, S2 combinations vary widely—for example, B-&CN performs well (MAE = 0.254), but B&CM performs poorly (MAE = 1.820)—highlighting that the predictive success of superspectra depends critically on substrate chemistry rather than the number of concatenated spectra.

To comprehensively visualize the model performance across all substrate combinations, we constructed a two-dimensional error plot with MAE and RMSE as complementary metrics. Because typical regression models in this study exhibit RMSE values of approximately √2× MAE, we scaled MAE by √2 to place both metrics on comparable scales

1 $$\begin{array}{l} \mathrm{weighted}\;\mathrm{MAE}=\sqrt{2}\times \mathrm{MAE} \end{array}$$

Figure 6 plots weighted MAE (*x*-axis) against the RMSE (*y*-axis) for all RFR model configurations. The radius of the contour circles represents constant combined error, defined as

2 $$\begin{array}{l} r=\sqrt{\mathrm{weighted}\;\mathrm{MA}E^{2}+\mathrm{RMS}E^{2}}=\sqrt{2\times \mathrm{MA}E^{2}+\mathrm{RMS}E^{2}} \end{array}$$

Points closer to the origin (smaller *r*) indicate a superior predictive performance. Three reference contours (*r* = 0.75, 2.0, 3.35) are shown to guide the visual assessment. The following key observations can be found from Figure 6: (1) The S2 superspectrum CM&CN performs best, appearing closest to the plot origin with the lowest weighted MAE and RMSE. (2) The S3 combination B&CM&CN also shows excellent performance and ranks second overall. (3) Some S2 and S3 combinations outperform the four-substrate S4 case, demonstrating that more complex superspectra do not necessarily yield better predictions. (4) Combinations including MCH (e.g., B&CM&MCH, S4) show degraded performance due to noninformative or conflicting spectral contributions from the negatively charged MCH surface. These trends further reinforce a central conclusion of this study: the effectiveness of superspectra depends on the chemical complementarity of substrates, not on the total number of substrates concatenated.

**Figure 6:**
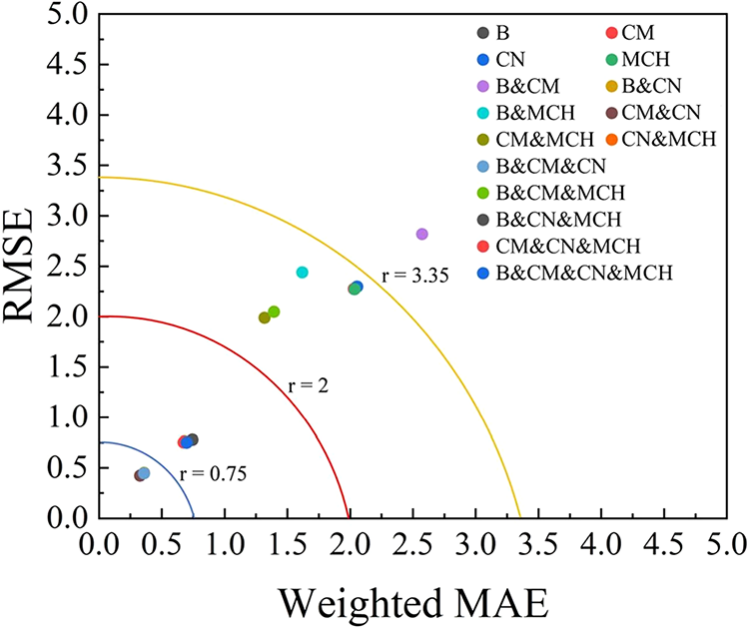
Weighted MAE vs RMSE for all S1–S4 superspectra combinations using the RFR model. Each colored point represents a specific substrate combination. Contour curves indicate performance thresholds, with points closer to the origin representing lower prediction error. Combinations within smaller contour regions demonstrate superior predictive accuracy.

To further evaluate model robustness, we performed the same analysis using SVR (see Figures S4–S9 **)**. Although SVR provided reasonable predictions for some combinations, its overall performance was worse than RFR. As summarized in Table S4 and visualized in Figure S9, SVR results cluster at significantly higher weighted MAE (>3) and higher RMSE (>4). Correspondingly, *R* ^2^ values are consistently lower, showing a reduced predictive stability. These outcomes demonstrate that SVR struggles to generalize across multisubstrate superspectra, whereas RFR consistently achieves higher accuracy and robustness.

The superior performance of the S2 CM&CN and S3 B&CM&CN superspectra combinations can be explained by the complementary interaction modes between BSA and each substrate. CM provides strong electrostatic attraction through its positively charged −NH_3_^+^ group and introduces characteristic vibrational modes (e.g., 632 and 1136 cm^–1^) that encode specific chemical interactions with negatively charged BSA residues. CN, in contrast, is zwitterionic and interacts more evenly with both acidic and basic residues. Its signature 1047 cm^–1^ mode reflects its unique C−α–N environment and its ability to modulate BSA adsorption through changes in residue orientation and conformational constraints. When combined, CM stabilizes BSA near the SERS enhancement region, while CN provides additional, nonredundant chemical information—producing a superspectrum that captures orthogonal aspects of BSA–surface interactions.

The S3 combination (B&CM&CN) further enhances prediction robustness by adding spectra from the unmodified B substrate, which captures nonspecific adsorption and serves as a neutral reference. The integration of three distinct interaction modes, neutral (B), cationic (CM), and zwitterionic (CN), broadens the spectral representation of BSA, reduces systematic biases from any single surface chemistry, and produces one of the most accurate prediction models in this study.

In contrast, adding MCH (negatively charged) to the S4 combination degrades the predictive performance. Because MCH repels BSA’s acidic residues, it generates spectra with weak or missing protein peaks. When concatenated into superspectra, these noninformative or conflicting signals dilute the chemically meaningful features provided by B, CM, and CN. Thus, the reduced performance of S4 underscores that effective superspectra require chemically complementary substrates—not simply more substrate types.

Across all S1–S4 combinations, two general design principles emerge for superspectra: (1) Chemical complementarity is essential. The best-performing combinations (CM&CN and B&CM&CN) pair substrates with distinct charge properties and interaction modes that generate nonredundant spectral information. (2) More substrates do not guarantee better performance. S4 combinations incorporating MCH perform worse despite using more data, demonstrating that substrate incompatibility can overwhelm the benefits of increased spectral diversity.

Although this study examines BSA under controlled aqueous conditions, real biofluids (e.g., serum, plasma, saliva, and urine) contain complex mixtures of proteins, metabolites, lipids, and salts that compete for adsorption and jointly shape the SERS response. In such environments, the “protein concentration” measured by SERS reflects a composite mixture rather than a single analyte. Importantly, the interaction modes captured by different functionalized surfaces extend beyond simple electrostatic effects. Chemical, electrochemical, and physical perturbations introduced by surface modifiers may alter the conformation, orientation, or binding state of entire protein populations or selectively affect subsets of proteins. By integration of spectra collected under these diverse interaction conditions, superspectra can expand the molecular information space, enabling machine learning models to extract latent chemical signatures from complex mixtures.

## IV Conclusion

In this work, we investigated the SERS response of BSA proteins adsorbed onto Ag nanorod substrates functionalized with three thiol modifiers—cysteamine (CM), cysteine (CN), and MCH—and demonstrated how substrate-dependent spectral variations can be leveraged through superspectra integration to substantially improve quantitative protein analysis. As expected, surface chemistry strongly modulates protein adsorption behavior and vibrational signatures, but our results show that these modulation effects can be exploited rather than treated as sources of variability. By concatenating spectra from chemically complementary substrates into superspectra, we were able to enhance the information richness of the data and significantly improve the quantitative prediction accuracy.

Initial analyses of peak intensities across BSA concentrations revealed highly nonmonotonic behavior, particularly on modified substrates, confirming the limitations of traditional calibration-curve approaches. Machine learning was, therefore, used to extract quantitative information from the full spectral patterns. Classification models (SVM and RF) achieved 100% accuracy in distinguishing the four substrate types, confirming that each surface chemistry produces a distinct spectral signature and validating the central premise of superspectra: concatenating spectra from different substrates introduces complementary rather than redundant features.

For concentration prediction, regression models (SVR and RFR) were evaluated across all single-substrate and superspectra combinations. RFR consistently outperformed SVR in accuracy, stability, and generalizability. The best-performing configurations, CM&CN (S2) and B&CM&CN (S3), yielded 4–5 times reductions in prediction error relative to single-substrate models (MAE reduced from 1.44 to 0.25 mg/mL; *R* ^2^ improved from 0.795 to 0.993). These gains arise not from the number of concatenated spectra but from the complementarity in substrate chemistry: CM provides strong cationic interactions, CN introduces balanced zwitterionic interactions, and B offers a neutral reference baseline. Conversely, combinations including MCH (negatively charged) degraded performance due to weak or repulsive protein interactions, demonstrating that substrate compatibility, not substrate count, determines superspectra quality.

This study’s primary contribution is establishing an understanding of the design principle of chemically complementary superspectra for quantitative protein SERS. By integrating spectra from substrates with orthogonal interaction modes, superspectra capture a broader representation of protein–surface interactions and produce markedly improved quantitative predictions. This framework provides a foundation for extending superspectra-guided SERS to more complex biomolecular systems.

Despite the significant improvements demonstrated in this study, several important challenges remain before the superspectra strategy can be fully generalized or translated into real-world protein sensing. First, although our empirical testing identified CM&CN and B&CM&CN as optimal combinations for BSA, a broader mechanistic framework is still needed to guide substrate selection for other proteins. Predictive models that relate protein physicochemical properties such as charge distribution, isoelectric point, hydrophobicity, and structural flexibility to optimal substrate chemistries would allow superspectra design to shift from exhaustive empirical screening to theory-driven optimization.

A second major challenge is the need to validate this approach in real biological fluids. Unlike pure BSA solutions, serum, plasma, urine, and saliva contain diverse proteins, lipids, metabolites, and salts that compete for adsorption and collectively shape the SERS response. These complex matrices introduce both competitive binding effects and spectral overlap, making quantitative prediction more difficult. However, the superspectra framework may help disentangle these mixed spectral contributions. Systematic studies in real biofluids are essential to determine the generalizability and robustness of this strategy.

A third opportunity lies in extending the framework from single-analyte prediction to multianalyte quantification. Real diagnostic applications often require simultaneous measurement of several proteins (e.g., albumin, immunoglobulins, and C-reactive protein). The high-dimensional, chemically diverse information encoded in superspectra may enable multioutput regression or latent-variable modeling capable of resolving multiple protein signatures from a single measurement. Demonstrating this capability would significantly broaden the diagnostic utility of the method.

Another important direction is mechanistic modeling. Although this study provides empirical insights into why certain substrate combinations are complementary, quantitative models linking surface properties such as charge density, functional group spacing, hydrophobicity, or SAM packing order to superspectral complementarity would greatly enhance our ability to design effective surface chemistries. Incorporating principles from electrostatics, protein adsorption theory, or molecular simulations could provide a physics-informed basis for the optimization of substrate combinations.

Finally, algorithmic improvements remain an open area for advancement. Expanding the data set size, refining model hyperparameters, and exploring advanced ML architectures such as attention-based models, graph neural networks, or latent-variable methods may further improve predictive accuracy and resilience to spectral complexity. Such developments will be essential for achieving strong generalization in real-world biosensing applications, where noise, variability, and matrix effects are unavoidable.

## Supplementary Material



## Data Availability

The code and data used in this manuscript are available at https://github.com/jimcui3/BSA-protein-superspectra.

## Notes

Detailed experimental setup, spectral variability analysis results, and additional machine learning experimental results (PDF)
