# Supplementary material for: Improving Protein Quantification with SERS Superspectra and Machine Learning
Source: ACS Omega. 2026 Feb 4;11(6):10843–53. doi: 10.1021/acsomega.6c00157 (PMC12917838; doi:10.1021/acsomega.6c00157)
Supplement: Supplementary file 1 [file ao6c00157_si_001.pdf]

Supporting Information

Improving Protein Quantification with SERS Superspectra and Machine Learning

Jiaheng Cui [a #], Chenyao Feng [b #], Xulan Chen [c], Yanjun Yang [d], Pengju Yin [b *], Yiping Zhao [d *]

[a] School of Electrical and Computer Engineering, College of Engineering, The University of Georgia, Athens, GA, USA 30602

[b] School of Mathematics and Physics, Hebei University of Engineering, Handan, Hebei, China 056038

[c] Department of Biochemistry and Molecular Biology, Franklin College of Arts and Sciences, The University of Georgia, Athens, GA, USA 30602

[d] Department of Physics and Astronomy, Franklin College of Arts and Sciences, The University of Georgia, Athens, GA, USA 30602

[#] These authors contributed equally to this work

[*] Corresponding Author: E-mail: yinpengju@hebeu.edu.cn, zhaoy@uga.edu

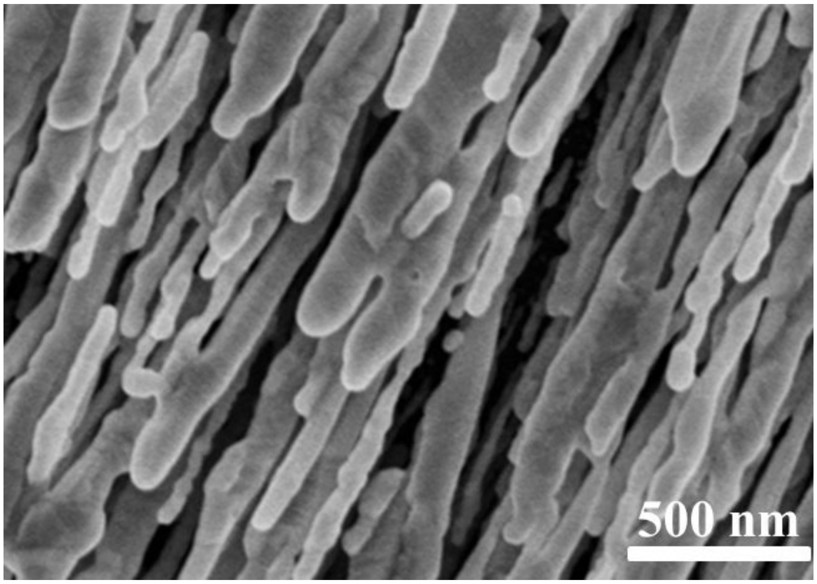

Figure S1. Representative SEM image of the AgNR substrate.

Section S1. Superspectra Construction and Combinatorial Spectral Enumeration

For a single-modification (S1) combination, the number of superspectra equals its own effective spectral count. The number of superspectra generated by an S2 combination is the product of the spectral counts of the two different substrates at the same BSA concentration. For example, at a concentration of 0.05 mg/mL, the B substrate has 112 spectra and the CM substrate has 52 spectra; thus, the number of superspectra for the B connected to CM (B&CM) combination is 112 × 52 = 5,824. The number of superspectra generated by an S3 combination is the product of the spectral counts of the three different substrates at the same BSA concentration. For example, at a concentration of 0.05 mg/mL, the B substrate has 112 spectra, the CM substrate has 52 spectra, and the CN substrate has 51 spectra; thus, the B connected to CM connected to CN (B&CM&CN) combination yields a total of 112 × 52 × 51 = 297,024. The number of superspectra generated by an S4 combination is the product of the spectral counts of the four different substrates at the same BSA concentration. For example, at a concentration of 0.05 mg/mL, the B substrate has 112 spectra, the CM substrate has 52 spectra, the CN substrate has 51 spectra, and the MCH substrate has 53 spectra; thus, the number of superspectra for the B connected to CM connected to CN connected to MCH (B&CM&CN&MCH) combination is 112 × 52 × 51 × 53 = 15,742,272. For the B substrate, 120 spectra were measured at each concentration. For the other three modified substrates, 60 spectra were measured at each concentration. After data preprocessing, some outliers were discarded. All superspectra counts are shown in **Table S1**.

Table S1. Number of spectra for all substrate combinations at different BSA concentrations.

SERS Substrates Concentration (mg/mL)	0	0.05	0.001	2×10^{-5}	4×10^{-7}	8×10^{-9}
**B**	60	112	107	114	113	108
**CM**	30	52	48	52	54	60
**CN**	30	51	49	49	52	49
**MCH**	30	53	56	58	57	55
**B&CM**	/	5824	5136	5928	6102	6480
**B&CN**	/	5712	5243	5586	5876	5292
**B&MCH**	/	5936	5992	6612	6441	5940
**CM&CN**	/	2652	2352	2548	2808	2940
**CM&MCH**	/	2756	2688	3016	3078	3300
**CN&MCH**	/	2703	2744	2842	2964	2695
**B&CM&CN**	/	297024	251664	290472	317304	317520
**B&CM&MCH**	/	308672	287616	343824	347814	356400
**B&CN&MCH**	/	302736	293608	323988	334932	356400
**CM&CN&MCH**	/	140556	131712	147784	160056	161700
**B&CM&CN&MCH**	/	15742272	14093184	16847376	18086328	17463600

Table S2. Hyperparameter ranges tested during grid search for RFR and SVR regression models.

Model	Kernel/Structure	Hyperparameters Tested
**SVR**	Linear	$C \in [10^{-3}, 10^{-2}, 10^{-1}, 1, 10, 100, 1000]$
	Polynomial	degree $\in [3, 5, 7, 9, 11]$
	RBF	$C \in [10^{-3}, 10^{-2}, ..., 10^{5}]$<br>$\epsilon \in [10^{-4}, 10^{-3}, ..., 10^{2}]$
**RFR**	Decision Trees	n_estimators $\in$ [100, 1000, 10000]<br>max_depth $\in$ [6, 10, None]

Section S2. Spectral variability analysis results.

The detailed spectral features described for **Figure 3** are based on average normalized spectra, which raises the question of measurement reproducibility across replicates. To validate the averaging approach and assess spectral consistency, we evaluated within-group variability for each (modification, concentration) combination. **Figure S2** presents representative examples at the highest (0.05 mg/mL) and lowest (8×10^{-9} mg/mL) BSA concentrations for all four substrate modifications. Each panel shows the average spectrum (solid line) overlaid with ±1 standard deviation shading (shaded region) calculated across all replicates within that condition. The narrow standard deviation bands and consistent peak positions across replicates demonstrate that within-group spectral variation is modest, validating the use of averaged spectra in **Figure 3** for comparative analysis.

Quantitatively, we calculated the average relative standard deviation (RSD) across all wavenumbers (401–1799 cm^{-1}) for each substrate modification. The RSD values, averaged across all BSA concentrations, are 9.2 ± 8.6% for B, 5.7 ± 6.2% for CM, 4.2 ± 6.5 % for CN, and 6.0 ± 8.1% for MCH, confirming good measurement reproducibility. These RSD values indicate that the peak positions and intensity patterns discussed above are reproducible features rather than measurement artifacts. The averaging strategy employed in **Figure 3** effectively captures consistent spectral features while suppressing random noise, enabling reliable identification of BSA-specific and modification-specific vibrational modes.

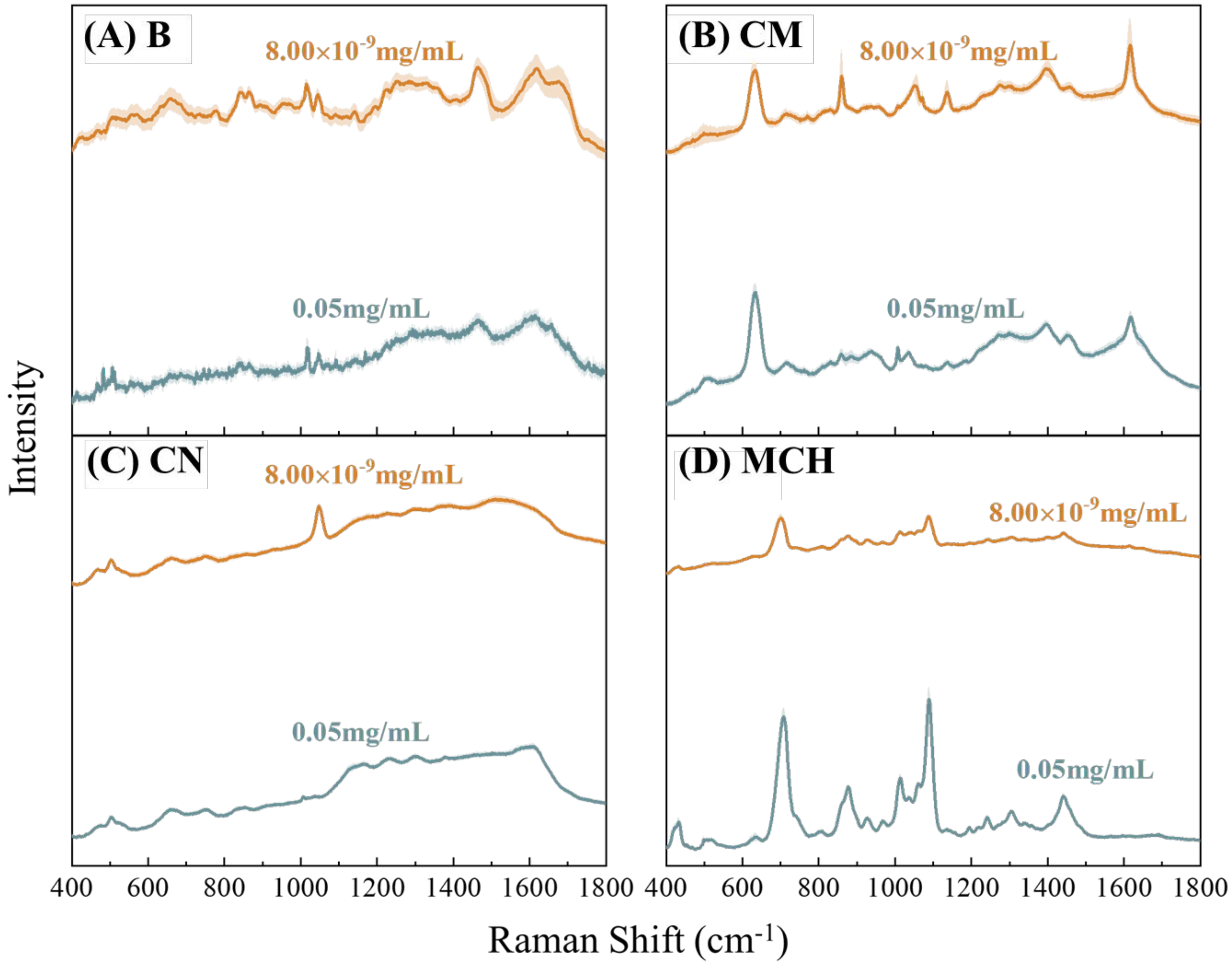

Figure S2. Representative spectral variability within measurement groups. SERS spectra for four substrate modifications at the highest (0.05 mg/mL, blue) and lowest (8×10^{-9} mg/mL, orange) BSA concentrations, showing individual replicates overlaid with group statistics. (A) Bare substrate, (B) Cysteamine-modified, (C) Cysteine-modified, (D) MCH-modified. In each panel, solid lines represent the average normalized spectrum, and shaded regions indicate ±1 standard deviation calculated across all replicates within that (modification, concentration) group.

Section S3. Additional machine learning experimental results

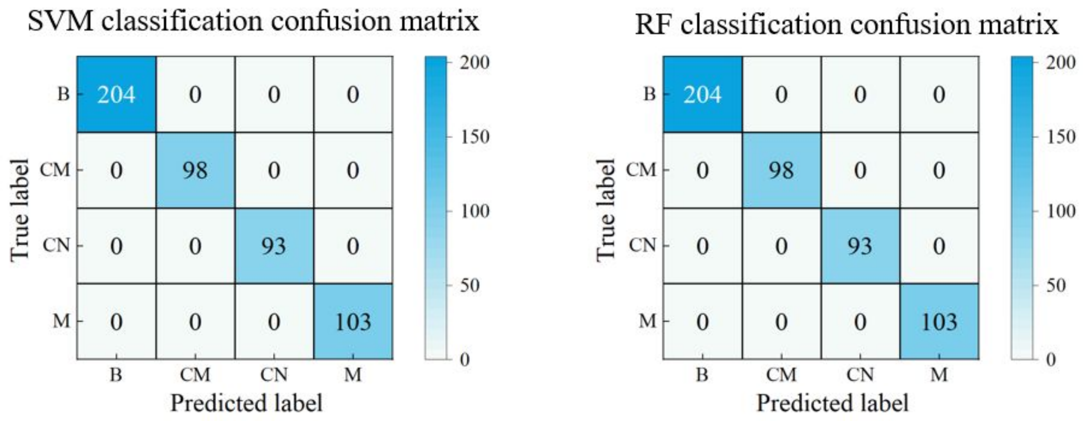

Figure S3. Confusion matrices for SVM and RF classifiers distinguishing four substrate modification types (B, CM, CN, MCH). Both models achieved 100% classification accuracy.

Table S3. Performance metrics (MAE, RMSE, R^2) for all substrate combinations (S1, S2, S3, S4) using RFR regression models.

**Result modification**	**S1**				**S2**						**S3**				**S4**
	B	CM	CN	M	B & CM	B & CN	B & M	CM & CN	CM & M	CN & M	B & CM & CN	B & CM & M	B & CN & M	CM & CN & M	B & CM & CN & M
**MAE**	1.443	0.484	0.477	0.481	1.820	0.254	1.145	0.235	0.938	0.483	0.254	0.986	0.526	0.464	0.494
**RMSE**	2.298	0.908	0.916	0.910	2.816	0.450	2.439	0.444	2.015	0.792	0.446	2.047	0.776	0.769	0.748
**R^2**	0.795	0.967	0.966	0.966	0.690	0.992	0.757	0.993	0.817	0.975	0.993	0.825	0.976	0.977	0.978

Table S4. Performance metrics (MAE, RMSE, R^2) for all substrate combinations (S1, S2, S3, S4) using SVR regression models.

**Result modification**	**S1**				**S2**						**S3**				**S4**
	B	CM	CN	M	B & CM	B & CN	B & M	CM & CN	CM & M	CN & M	B & CM & CN	B & CM & M	B & CN & M	CM & CN & M	B & CM & CN & M
**MAE**	1.184	1.097	0.532	0.841	3.088	2.344	2.124	0.637	0.848	0.712	2.340	1.943	1.892	0.682	1.713
**RMSE**	1.799	1.439	0.701	1.292	4.635	3.473	3.445	0.858	1.234	1.032	3.271	2.965	2.712	0.998	2.385
**R^2**	0.874	0.921	0.982	0.932	0.183	0.560	0.532	0.973	0.940	0.958	0.611	0.654	0.710	0.961	0.775

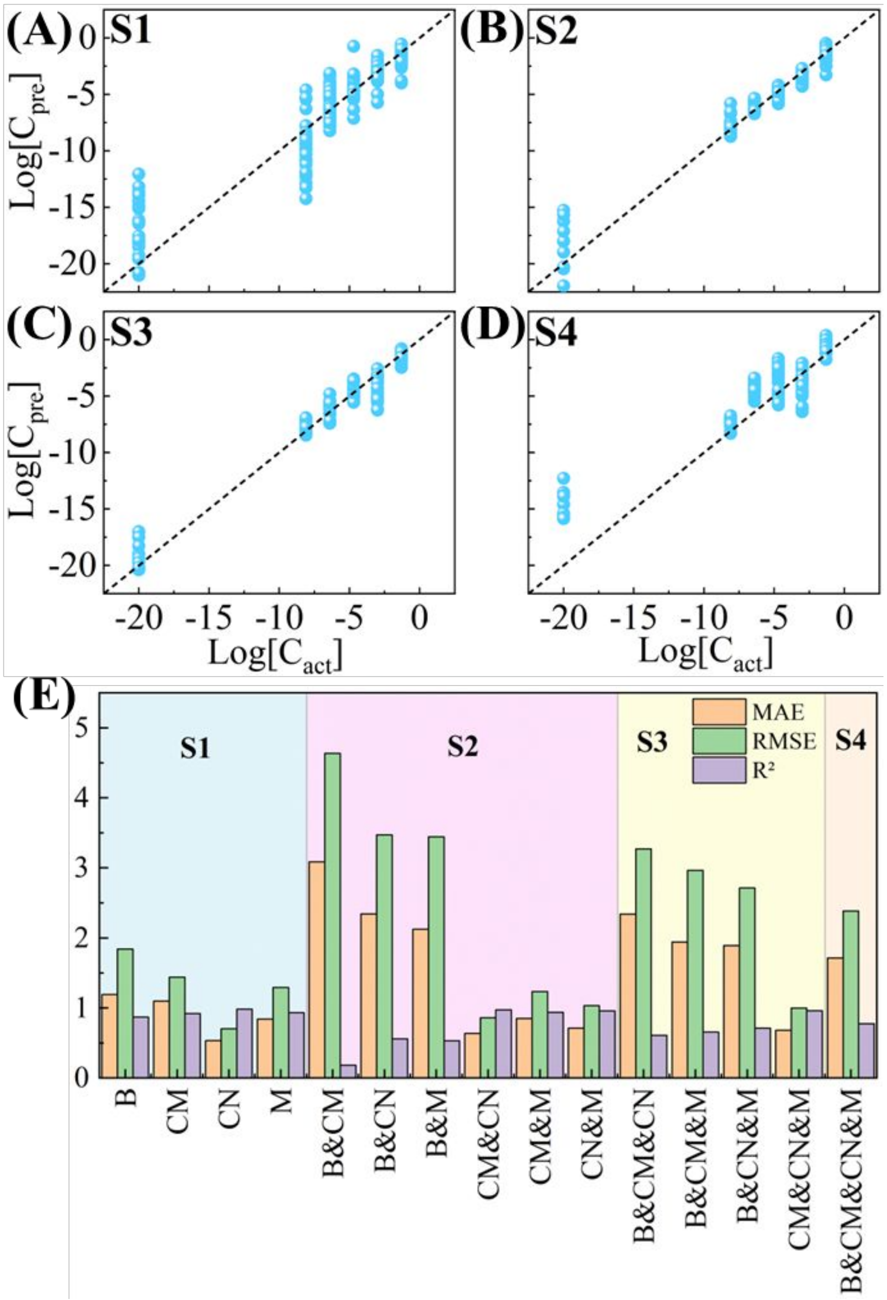

Figure S4. Predicted vs. actual BSA concentrations for best-performing SVR regression models in each superspectra category: (A) CN for S1; (B) CM&CN for S2; (C) B&CM&CN for S3; (D) B&CM&CN&MCH (the only S4 model). (E) Bar plots of MAE, RMSE, and R^2 for all substrate combinations.

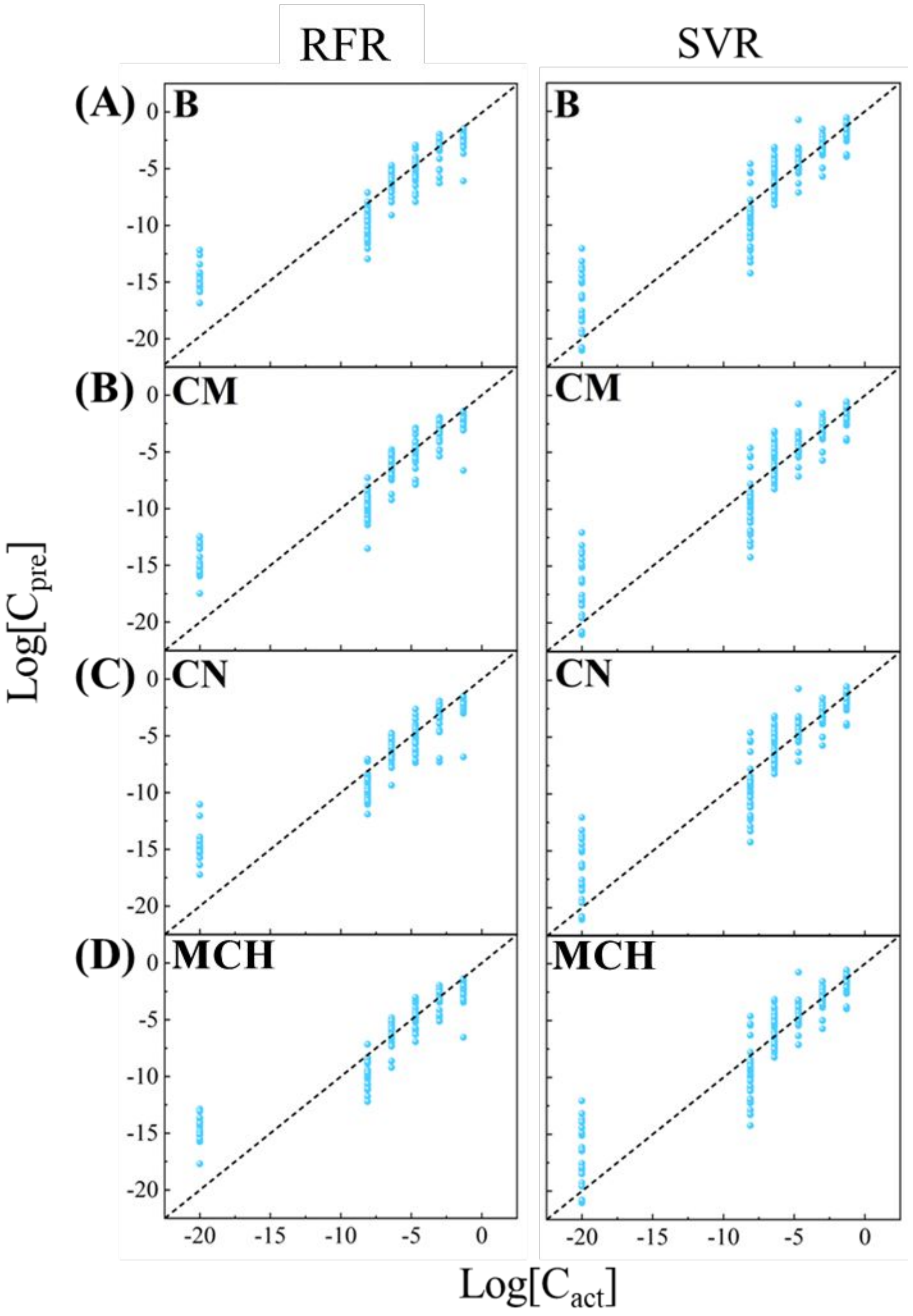

Figure S5. Predicted vs. actual BSA concentrations for all S1 combinations using RFR (left) and SVR (right) regression models.

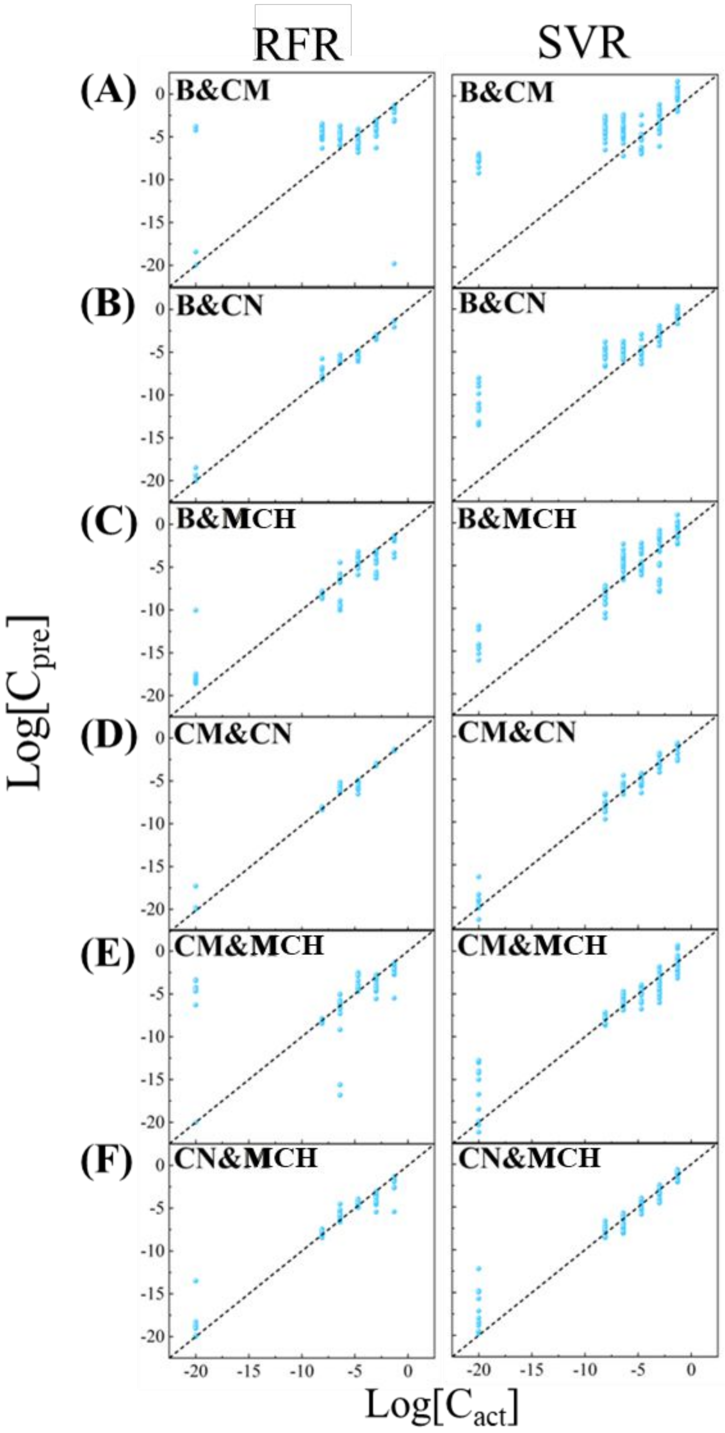

Figure S6. Predicted vs. actual BSA concentrations for all S2 combinations using RFR (left) and SVR (right) regression models.

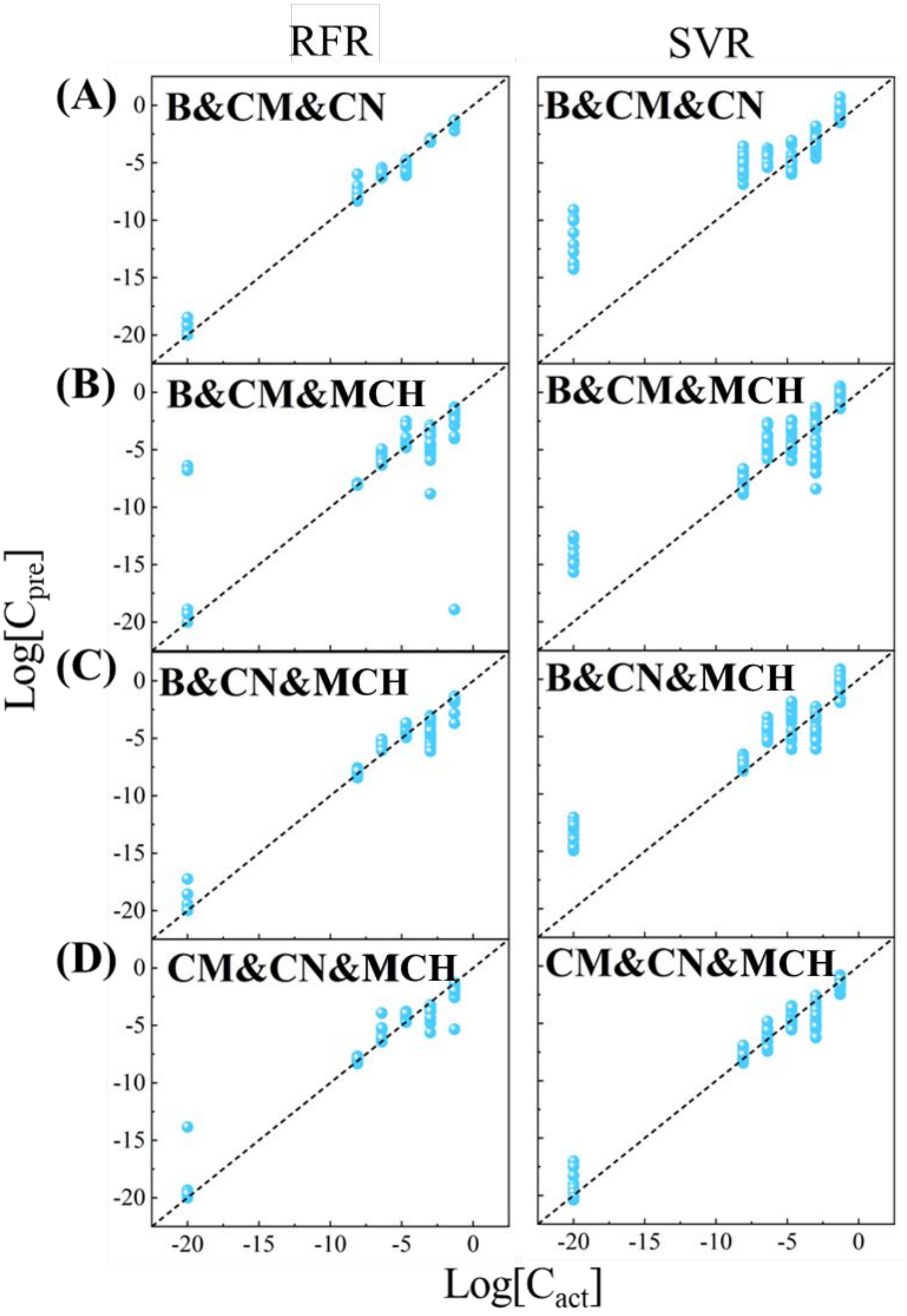

Figure S7. Predicted vs. actual BSA concentrations for all S3 combinations using RFR (left) and SVR (right) regression models.

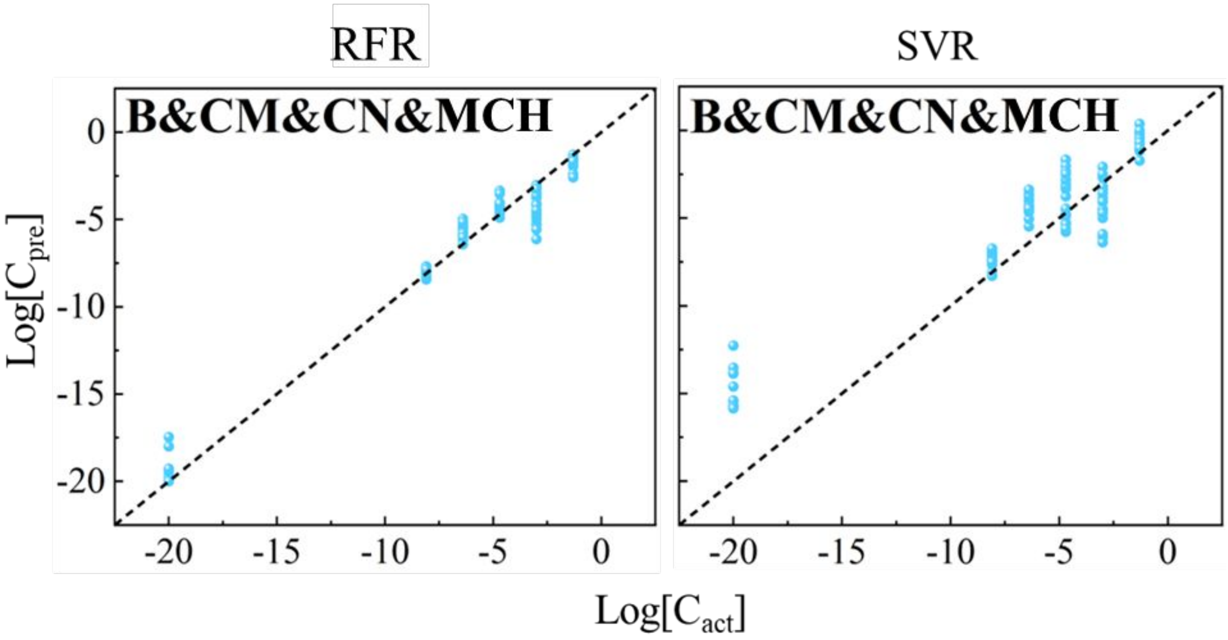

Figure S8. Predicted vs. actual BSA concentrations for the S4 combination using RFR (left) and SVR (right) regression models.

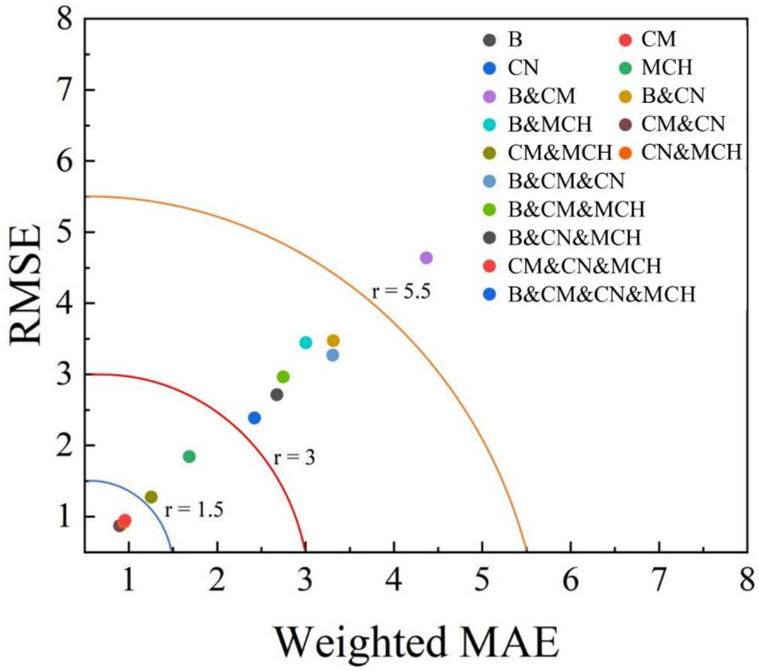

Figure S9. Weighted MAE versus RMSE for all S1–S4 superspectra combinations using the SVR model. Each colored point represents a specific substrate combination. Contour curves indicate performance thresholds, with points closer to the origin representing lower prediction error. Combinations within smaller contour regions demonstrate superior predictive accuracy.
